# Bilateral Gonadoblastoma in a 12‐Year‐Old Girl 4 Years After Wilms' Tumor: A Rare Pediatric Case

**DOI:** 10.1002/ccr3.72914

**Published:** 2026-06-09

**Authors:** Ahmed Quraish, Hasan Khouli, Yousef Daraghmeh, Sultan Mosleh, Samer Bustame

**Affiliations:** ^1^ Department of Scientific Research and Medical Projects, Faculty of Medicine and Allied Medical Sciences An‐Najah National University Nablus Palestine; ^2^ Department of Medicine, Faculty of Medicine and Allied Medical Sciences An‐Najah National University Nablus Palestine; ^3^ An‐Najah National University Hospital Nablus Palestine

**Keywords:** disorders of sex development, gonadoblastoma, pediatric oncology, secondary neoplasm, Wilms tumor

## Abstract

This case of a 12‐year‐old patient who developed bilateral gonadoblastoma 4 years after treatment for Wilms tumor highlights the importance of rigorous, risk‐adapted long‐term surveillance and careful evaluation of incidental gonadal findings, even in asymptomatic patients.

## 1 Introduction

Gonadoblastoma is a rare ovarian tumor made up of germ cells and gonadal stroma cells, resembling those of dysgerminoma and Sertoli tumors, respectively. It usually occurs in individuals with disorders of sex development, particularly in cases of gonadal dysgenesis. However, rare cases where gonadoblastoma developed in phenotypically normal females with normal karyotypes have been reported. Approximately 40% of patients diagnosed with gonadoblastoma present with bilateral involvement [1]. This tumor is considered a premalignant lesion that can potentially transform into a malignant germ cell. Histopathologic studies have demonstrated a stepwise progression from the benign gonadoblastoma pattern to overt dysgerminoma tumor [2].

Wilms tumor (WT), also known as nephroblastoma, is the most common primary renal tumor in children characterized by an embryonal kidney mass affecting approximately 650 children in the United States each year. WT presents a significant clinical challenge due to its potential for aggressive behavior and metastasis [3]. Primary treatment typically involves a combination of surgical intervention, chemotherapy, and radiation therapy, tailored according to the disease stage at diagnosis [3, 4].

Despite advances in therapy, WT survivors are at risk of developing secondary malignant neoplasms (SMNs). Long‐term follow‐up guidelines for childhood cancer survivors highlight the necessity for organized, lifetime surveillance to detect and manage such issues because these late effects can appear years after completion of treatment [5, 6].

This report discusses a 12‐year‐old girl who developed a bilateral gonadoblastoma 4 years after being diagnosed with WT. The bilateral gonadoblastoma in this patient, identified through imaging and surgery, highlights the importance of extended Wilms tumor follow‐up. This case underscores the need for vigilance in monitoring WT survivors for late‐onset complications.

## 2 Case History/ Examination

A chronological summary of the patient's clinical course is presented in Table 1.

**TABLE 1:** Chronological summary of the patient's clinical presentation, diagnostic investigations, treatment interventions, and follow‐up from initial diagnosis of Wilms tumor at age 8 years to diagnosis of bilateral gonadoblastoma at age 12 years.

Age	Clinical event	Key finding	Management/outcome
8 years	Initial presentation	Left renal mass suggestive of Wilms' Tumor	Left radical nephrectomy with lymph node dissection, pathology consistent with Wilms' Tumor
8 years (post‐op)	Adjuvant therapy	Completed chemotherapy over 18 months	Achieved clinical remission
9 years	Acute appendicitis (interim presentation)	Ultrasonography findings: noncompressible appendix with a fecalith, and incidental bilateral ovarian enlargement with multiple cysts	Appendectomy with mesenteric lymph node biopsy, no gynecologic follow‐up at that time
12 years	Recurrent abdominal pain	Ultrasonography and MRI findings: large midline cystic mass, bilateral ovarian enlargement with multiple cysts	Ovarian torsion was found, laparatomy was done, with ovarian biopsy.
12 years (1 week later)	Diagnosis of bilateral gonadoblastoma	Histopathology suggestive of gonadoblastoma	Left salpingo‐oophrectomy and right oophrectomy

An 8‐year‐old female presented to the urology outpatient clinic in a tertiary care hospital complaining of abdominal pain, urinary urgency, and nocturnal enuresis of 14 days duration. There was no history of dysuria, hematuria, urinary frequency, or fever. The patient has a known history of recurrent urinary tract infections since she was 3 years old and was treated on multiple occasions without any improvement. Physical examination revealed a nontender, soft abdomen with a palpable mass on the left. Doppler ultrasonography (U/S) of the abdomen showed a heterogeneous mass arising from the upper pole of the left kidney and into the left renal pelvis with internal vascular flow. Following this finding, an abdominal contrast‐enhanced computed tomography (CT) scan was performed. The contrast‐enhanced CT scan showed a well‐defined, lobulated left renal soft tissue mass with cystic changes occupying the upper pole of the kidney and bulging into the left renal pelvis, resulting in mild dilation of the left calyceal system measuring exactly 8 × 7.5 × 7.8 cm radiologically suggestive of a Wilms tumor (Figure 1).

**FIGURE 1:**
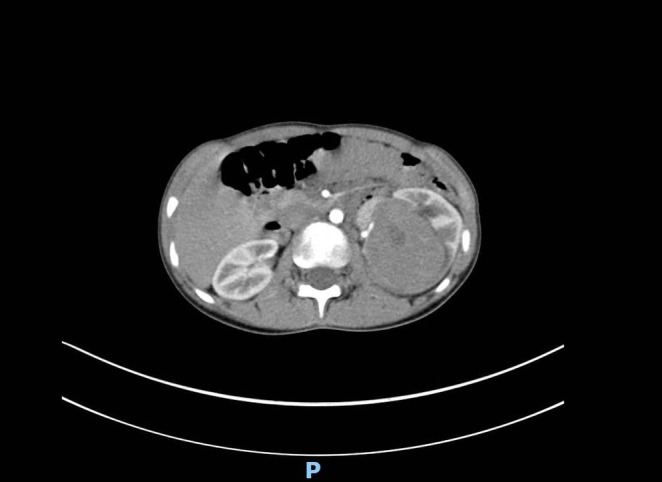
A contrast enhanced axial computed tomography (CT) scan of the abdomen of an 8‐year‐old female patient with Wilms Tumor before treatment, demonstrating a loculated soft tissue mass in the upper left kidney pole consistent with nephroblastoma.

Two weeks after the initial presentation with a primary diagnosis of Wilms tumor, the patient underwent a left radical nephrectomy with bilateral para‐aortic lymph node dissection. Histopathology was consistent with Wilms tumor. Histopathologic subclassification was not available in the archived pathology reports. Eight days after the surgery, the patient was started on the National Wilms Tumor Study (NWTS) (COG) protocol. The chemotherapy protocol was continued for 18 weeks, and it was recommended that the patient undergo a positron emission tomography (PET) scan 3 weeks after the last chemotherapy session.

At age 9, 7 months after the last chemotherapy session, the patient presented to the emergency department (ED) complaining of severe and sharp right lower quadrant abdominal pain of 4‐h duration. The pain was associated with fever, anorexia, nausea, and dysuria. On physical examination, the patient was febrile, with positive rebound tenderness in the right lower quadrant. Initial laboratory examination showed an elevated white blood cell count and C‐reactive protein. Subsequently, an abdominal US was done, and it revealed a noncompressible appendix, measuring 6 mm in diameter with a fecalith inside. Abdominal US also showed enlarged mesenteric lymph nodes and bilaterally enlarged ovaries with multiple cysts. Consequently, the patient underwent an appendectomy with mesenteric lymph node biopsy. Given the patient had no specific gynecologic symptoms and the acute surgical priority of appendicitis, the ovarian enlargement was considered incidental at the time and no immediate gynecologic follow‐up was done in regard to the enlarged ovaries following the surgery. Outpatient gynecologic follow‐up was advised; however, no further evaluation was documented until the subsequent presentation at age 12.

At 12 years of age, the patient presented to the ED complaining of sharp right lower quadrant abdominal pain of 4 days duration, which became severe in the 24 h preceding the presentation. There was no history of diarrhea or constipation, vomiting, dysuria, hematuria, urinary frequency, or menstrual abnormalities. On physical examination, the patient looked ill, in pain, and was febrile. There was notable right lower quadrant tenderness on abdominal examination.

## 3 Differential Diagnosis, Investigations, and Treatment

Following clinical examination, abdominal ultrasound revealed a large midline pelvic cyst measuring 8.2 × 4.4 cm with internal echogenic debris and thick wall, with bilateral ovarian enlargement containing innumerable tiny follicles with preserved vascularity. A small amount of pelvic free fluid was also noted in the US. Subsequently, pelvic magnetic resonance imaging (MRI) was ordered, and it revealed a large midline simple cystic mass measuring 9.2 × 6 × 6.3 cm. It also showed significantly enlarged right ovary with a nonenhancing central area measuring 7.5 × 3.5 × 8 cm that extends into the pouch of Douglas. The left ovary was also enlarged to a lesser extent measuring 3 × 4.2 × 5.5 cm. Both ovaries had innumerable small sub‐centimeter peripherally enhancing cysts (Figure 2A,B). Following the MRI, a laparotomy was scheduled and tumor markers were drawn. During the procedure, peritoneal lavage was done, and right‐sided ovarian cyst torsion with fallopian tube ischemia were identified. Consequently, the large midline cyst and right fallopian tube were excised. A biopsy of the left ovary was also performed during the same procedure. One week later, the histopathology report was issued suggesting bilateral gonadoblastoma. Histopathological images and slides were not available in the archived pathology data. The tumor markers revealed normal results: LDH: 145, CEA: 1.24, CA 15–3: 7.73, CA 19–9: 6.4, AFP: 1.48, B‐HCG: 0.1. After that, a left salpingo‐oophorectomy and a right oophorectomy were performed. Incidentally, Meckel's diverticulum was discovered intraoperatively and resected. Following the surgery, chemotherapy treatment plan was scheduled but was not given.

**FIGURE 2:**
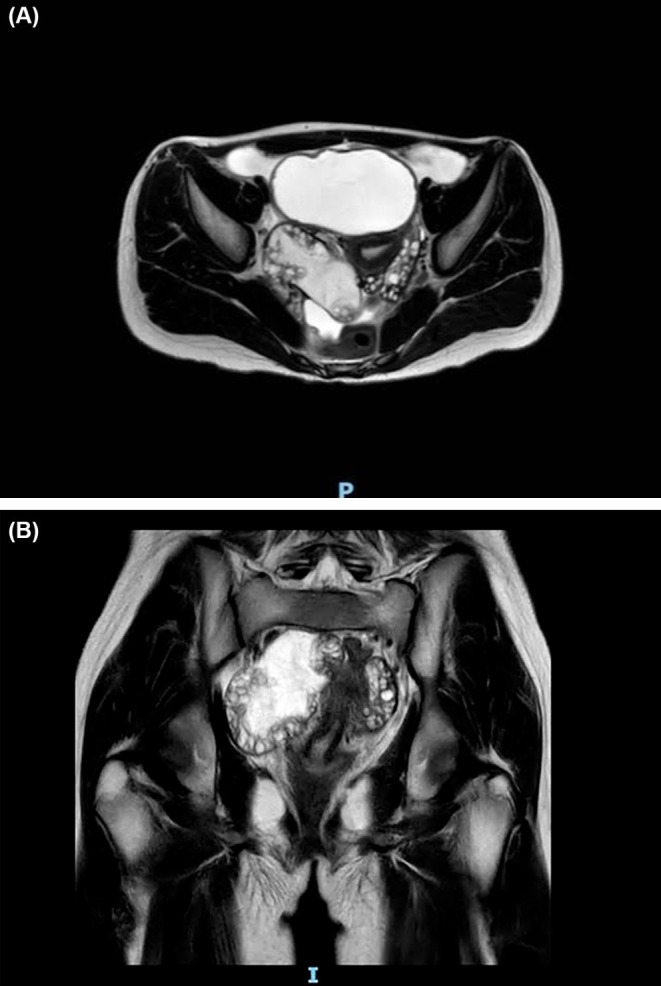
A T2‐weighted axial (A) and coronal (B) magnetic resonance imaging (MRI) scan of the abdomen of a 12‐year‐old female patient, demonstrating a large midline cystic mass with bilateral cystic changes in the ovaries consistent with bilateral gonadoblastoma prior to surgical intervention.

## 4 Results and Follow‐Up

As a result, hormonal workup was done and revealed normal values regarding menstrual phase: FSH: 0.3, LH: 1.57, 17‐B estradiol: 65.3. During her stay in the pediatric Intensive care unit (ICU), a full systemic evaluation was conducted and revealed the presence of an atrial septal defect. During a follow‐up visit 8 weeks later, the patient was examined by an endocrinologist and was found to have orthostatic hypotension, breast tissue tanner Stage 3, axillary hair tanner Stage 2, pubic hair tanner Stage 2, and mild clitoral hypertrophy. A genetic analysis was also recommended; however, it was not performed at the time of writing due to the lack of financial coverage.

## 5 Discussion

Alongside neuroblastoma and hepatoblastoma, Wilms tumor is among the three most common pediatric abdominal malignant tumors [7]. Gonadoblastoma, however, is an inherently rare tumor, as it occurs mainly in individuals with disorders of sex development [8]. This case report discusses a 12‐year‐old female patient who was diagnosed with bilateral gonadoblastoma 4 years after completing treatment for Wilms tumor. This subsequent occurrence of bilateral gonadoblastoma is considered a rare phenomenon that raises significant clinical considerations. Ovarian or gonadal neoplasms following Wilms tumor have only been reported in a few isolated case reports. Notably, Ansari‐Damavandi et al. reported a secondary ovarian germ cell tumor that developed 8 years following Wilms tumor treatment. In that case, the ovarian cancer was unilateral and developed as a late complication in a long‐term survivor [9]. There have also been reports of rare associations between Wilms tumor and ovarian involvement, such as a case of concurrent Wilms tumor affecting the kidney and ovary [10]. In contrast, the present case is distinguished by the development of bilateral gonadoblastoma, a premalignant gonadal lesion, 4 years following completion of Wilms tumor therapy. This highlights the significance of structured follow‐up when gonadal abnormalities are found in Wilms tumor survivors. To our knowledge, there are no previously reported cases describing the development of bilateral gonadoblastoma following completion of Wilms tumor resolution.

The complex nature of this case posed significant diagnostic and therapeutic challenges in regards to the patient's clinical presentation and medical history. The patient's presentation with abdominal pain could have been attributed to a variety of causes. Additionally, the patient's medical history of Wilms tumor further complicated the diagnostic evaluation. Therefore, radiologic studies remain a cornerstone in evaluating abdominal pain. Despite that, imaging can still provide inconclusive results especially in those with complicated medical history, as in this case. Furthermore, the ischemic changes encountered during surgery emphasize the importance of early detection of gonadoblastoma and its complications especially in those with a history of Wilms tumor. The incidental discovery of bilateral ovarian enlargement during the appendectomy at age nine is a crucial learning point from this case. Despite the recommendation for an outpatient gynecologic follow‐up, no more evaluation was recorded until the patient's subsequent presentation. This emphasizes how difficult it is to maintain continuity of care following incidental findings, especially for pediatric cancer patients. In order to avoid delayed detection and associated complications in this population, even asymptomatic gonadal abnormalities may warrant coordinated follow‐up pathways and surveillance imaging.

Wilms tumor occurrence has been associated with at least 100 syndromes [11]. Of all Wilms tumor cases, 9%–17% are associated with underlying syndromes, such as WAGR (Wilms–Aniridia–Genitourinary–mental retardation), Beckwith–Weidmann syndrome (BWS), Frasier syndrome (FS), Denys–Drash syndrome (DDS) [12]. A tumor suppressor gene that is linked with many of these syndromes is the WT1 gene located on chromosome 11p13. A mutation in this gene can be a direct cause of disorders of sexual development and gonadal malignancies [13]. Gonadoblastoma is a gonadal neoplasm containing multiple types of germ cells, such as granulosa and Sertoli cells. As mentioned previously, gonadoblastoma predominantly occurs in individuals with conditions related to disorders of sex development [14]. A related report described early bilateral gonadoblastomas in a patient with DDS, a WT1 mutation‐associated disorder characterized by nephropathy and gonadal dysgenesis. This highlights the potential for early gonadal neoplasia in the setting of genetic predisposition [15]. Although genetic syndromes such as DDS were suspected, genetic testing and karyotyping were not performed due to financial constraints. The lack of karyotyping and genetic testing in this case is a major limitation because a chromosomal abnormality or WT1‐related disease may have offered a unique and definitive pathologic explanation for the development of both bilateral gonadoblastoma and Wilms tumor. Nonetheless, this case raises the possibility of an underlying genetic susceptibility that remained clinically unrecognized.

Other than genetic factors, treatment‐related factors may also contribute to the development of secondary neoplasms in Wilms tumor survivors. Chemotherapy and radiation used in pediatric oncology can have DNA‐damaging effects. While these treatments target cancer cells, they unavoidably harm healthy cells, which can have serious adverse consequences on healthy tissues and organs [16]. Compared to the general population, pediatric cancer survivors are known to have a significantly higher incidence of therapy‐related secondary malignancies [17]. Moreover, over long‐term follow‐up periods, survivors treated according to National Wilms' Tumor Study protocols showed a rising cumulative incidence of secondary malignant neoplasms, which is indicative of the long‐term effects of multimodal therapy, such as chemotherapy and radiation [18]. Even though a direct causal association cannot be proven in this case, the possible interaction between previous chemotherapy exposure and underlying genetic predisposition deserves further investigation. Additionally, the absence of pathology reports and histopathological images represents a limitation of this case.

## 6 Conclusion

This case emphasizes the significance of long‐term, risk‐adapted surveillance for Wilms tumor survivors. Even in patients who are asymptomatic, incidental gonadal abnormalities discovered during interim clinical visits should not be disregarded. As part of long‐term surveillance, systematic gynecologic follow‐up and periodic pelvic imaging may be used for female Wilms tumor survivors, especially those with unusual results or suspected underlying genetic predisposition. Early multidisciplinary management may assist in preventing delayed diagnosis and related difficulties as well as enable prompt detection of subsequent neoplasms.

## Author Contributions

**Ahmed Quraish:** conceptualization, visualization, writing – original draft, writing – review and editing. **Hasan Khouli:** conceptualization, resources, visualization, writing – original draft, writing – review and editing. **Yousef Daraghmeh:** conceptualization, writing – original draft. **Sultan Mosleh:** conceptualization, project administration, resources, supervision, validation, visualization. **Samer Bustame:** conceptualization, project administration, resources, supervision, validation, visualization.

## Funding

The authors have nothing to report.

## Consent

Written informed consent was obtained from the patients' guardian for publication of this case report. This study was exempt from institutional review board (IRB) per institutional guidelines.

## Data Availability

The data that support the findings of this study are available from the corresponding author upon reasonable request.
